# Understanding CO adsorption in MOFs combining atomic simulations and machine learning

**DOI:** 10.1038/s41598-024-76491-x

**Published:** 2024-10-22

**Authors:** Goktug Ercakir, Gokhan Onder Aksu, Seda Keskin

**Affiliations:** Department of Chemical and Biological Engineering, Koç University, Rumelifeneri Yolu, Sariyer, 34450 Istanbul, Turkey

**Keywords:** Metal–organic framework (MOF), Carbon monoxide (CO), Adsorption, Molecular simulation, Machine learning, Chemical engineering, Computational chemistry, Atomistic models

## Abstract

This study introduces a computational method integrating molecular simulations and machine learning (ML) to assess the CO adsorption capacities of synthesized and hypothetical metal–organic frameworks (MOFs) at various pressures. After extracting structural, chemical, and energy-based features of the synthesized and hypothetical MOFs (hMOFs), we conducted molecular simulations to compute CO adsorption in synthesized MOFs and used these simulation results to train ML models for predicting CO adsorption in hMOFs. Results showed that CO uptakes of synthesized MOFs and hMOFs are between 0.02–2.28 mol/kg and 0.45–3.06 mol/kg, respectively, at 1 bar, 298 K. At low pressures (0.1 and 1 bar), Henry’s constant of CO is the most dominant feature, whereas structural properties such as surface area and porosity are more influential for determining the CO uptakes of MOFs at high pressure (10 bar). Structural and chemical analyses revealed that MOFs with narrow pores (4.4–7.3 Å), aromatic ring-containing linkers and carboxylic acid groups, along with metal nodes such as Co, Zn, Ni achieve high CO uptakes at 1 bar. Our approach evaluated the CO uptakes of ~ 100,000 MOFs, the most extensive and diverse set studied for CO capture thus far, as a robust alternative to computationally demanding molecular simulations and iterative experiments.

## Introduction

Carbon monoxide (CO) is a colorless, odorless, tasteless, and extremely poisonous gas, playing a crucial role in the carbon chemistry as a versatile feedstock, facilitating the catalytic transformation into diverse chemical products such as olefins, paraffins, alcohols, and aromatics^1–3^. CO has been also used as a selective reducing agent in several synthetic processes, and it holds promise in medical treatments^4,5^. Given its role in a variety of chemical and biological applications, effective storage of CO is critically important.

Metal–organic frameworks (MOFs) are considered as highly promising materials for efficient gas adsorption thanks to their tunable pore sizes, extensive surface areas, exceptionally high porosities, and customizable physical and chemical properties^6,7^. Recently, MOFs have been extensively studied for applications involving CO_2_, CH_4_, and H_2_ adsorption^8–10^. Although research on CO adsorption in MOFs has yielded promising results^11^, the number of studies in this area is very limited compared to studies on adsorption of other gases. This can be attributed to the nature of CO molecule: CO is toxic thus experimental measurement of CO adsorption is risky^12^, and molecular simulation of CO adsorption is computationally challenging due to the molecule’s polar nature which requires calculation of the electrostatic interactions between CO and MOFs in addition to the difficulty of identifying an accurate force field^11,13,14^.

In experimental studies, Deng and co-workers^15^ measured CO adsorption capacities of MOF-5 and MOF-177, and showed that MOF-177 can achieve the highest CO capacities (4.64 and 3 mol/kg at 194.5 and 237 K, respectively, at 1.08 bar), outperforming zeolites 5A and 13X. Li and co-workers^16^ synthesized MIL-100(Fe) and its Cu-doped versions, and measured their CO adsorption capacities in between 0.38–2.78 mol/kg at 1 bar, 298 K. Kim and co-workers^17^ followed a different approach to synthesize MIL-100(Fe) and its Cu-doped versions and their CO adsorption capacities were measured in between 0.26–3.10 mol/kg at 1 bar, 298 K. Petit and coworkers^18^ reported that CO adsorption of several MOFs (0.48–6.14 mol/kg) outperforms benchmark Cu-zeolite Y adsorbent (1.70 mol/kg) at 1 bar, 298 K.

Karra and Walton was first to utilize Grand Canonical Monte Carlo (GCMC) simulations and computed CO adsorption capacity of CuBTC as 11 mol/kg at 40 bar, 298 K^19^. They extended their approach to three other MOFs, IRMOF-1, IRMOF-3, Zn-MOF, and computed CO capacities up to ~ 12.5 mol/kg at 42.5 bar and 298 K^20^. Calero and co-workers^11^ utilized GCMC simulations to compute CO storage capacities of Cu-BTC, IRMOF-1, and MIL-47 at 298 K, between 1 and 10^3^ bar. Their results revealed that CO adsorption capacities up to ~ 11–18 mol/kg can be achieved at 10^3^ bar. Jorge and coworkers^13^ computed CO uptakes of HKUST-1 and IRMOF-1 in between ~ 0.2 and 0.8 mol/kg using various CO models at 1 bar, 298 K via GCMC simulations. Prakash and co-workers^21^ used GCMC simulations to compute CO capacities of ZIF-68 and ZIF-69 around 0.2 mol/kg at 1 bar, 298 K. Maurin and coworkers^22^ utilized GCMC simulations and showed that MIL-160(Al) achieves CO capacities up to ~ 4 mol/kg in between 0 and 50 bar, 303 K conditions. Wu and co-workers^23^ focused on two paddle-wheel MOFs and their chelated versions to compute CO adsorption capacities in between 10 and 110 cm^3^/g (corresponding to ~ 0.44–4.91 mol/kg) at 1 bar, 298 K. Yan and coworkers^24^ utilized GCMC simulations to compute CO adsorption capacities of two MOFs and two covalent organic frameworks (COFs) as 2.7–4.2 mol/kg at 10 bar, 298 K. Franz and coworkers focused on CO adsorption in Zn-MOF-470 and its functionalized versions using GCMC simulations^25^. Results revealed that hydroxyl (-OH) functionalized Zn-MOF-470 achieves the highest uptake at 116.2 cm^3^/g (~ 5.18 mol/kg) outperforming others at 1 bar, 298 K.

Limitless combinations of metals and organic linkers have resulted in a vast array of MOFs, with a recorded number of 125,383 distinct materials synthesized to date (as of May 2024)^26^, and these structures were collected in several MOF databases such as Computation-Ready and Experimental (CoRE) MOF Database^27,28^, and Cambridge Structural Database (CSD)^29,30^. Additionally, hundreds of thousands hypothetical MOFs (hMOFs) were computationally designed by mimicking the chemical and structural insights gathered from synthesized materials^31–35^. Given that experimental synthesis, characterization, and testing of each MOF for CO adsorption demand considerable resources, time, and effort; high-throughput computational screening (HTCS) of thousands of MOFs via molecular simulations hold immense potential. HTCS is invaluable not only for evaluating the CO adsorption potentials of a wide variety of materials, thereby guiding experimental efforts towards the most promising candidates, but also for revealing complicated relations between materials’ structures and their performances. However, HTCS studies for CO adsorption are very limited due to the computational difficulties that we mentioned above. Rampal and co-workers^36^ studied 183 CoRE MOFs using GCMC simulations to identify the MOFs with high CO capacities and CO/N_2_ separation potentials. The top MOFs were identified to have CO/N_2_ selectivities of 3.54–5.52 and CO working capacities of 0.82–6.21 mol/kg at 1–10 bar. Guo and co-workers^37^ screened 6301 porous materials including 4764 CoRE MOFs, 1200 hMOFs, 322 zeolites, and 15 COFs for CO adsorption using classical Density Functional Theory (cDFT) approach and identified the top adsorbents at 1 bar, 298 K conditions. Results revealed that top adsorbents are mostly MOFs and zeolites, and while being compatible to each other, they achieve high CO capacities of 3–4.4 mol/kg. These two HTCS studies highlighted the significant potential of MOFs compared to traditional porous materials for CO adsorption. However, these studies typically concentrate on a single MOF database, either real or hypothetical, which only encompasses a very small segment of the vast MOF landscape.

To fully exploit both synthesized and hypothetical MOFs for CO adsorption, we combined the powers of HTCS and machine learning (ML) in this work. We focused on two distinct MOF databases; CoRE MOF Database, which includes 14,142 synthesized structures; and a hypothetical MOF database containing 137,953 materials, offering a broad chemical variety. The CO adsorption data of CoRE MOFs were produced by performing HTCS and then this high-quality molecular data was used to train ML models to predict the CO adsorption capacities of very large numbers of hMOFs precisely and effectively. By integrating molecular simulations with ML, we analyzed a total of 100,364 different types of MOFs, the largest number of MOFs studied for CO adsorption to date, at different pressures 0.1, 1, 10 bar at room temperature. After analyzing the CO adsorption data of all MOFs, we pinpointed the most promising candidates from the synthesized and hypothetical materials and highlighted the essential chemical and structural features of the best MOFs offering high CO uptakes. Our computational approach will accelerate the design of new MOFs having improved CO adsorption capacities and act as an efficient alternative to labor-intensive experimental methods and exhaustive computer simulations for evaluating CO adsorption in crystal porous materials.

## Computational details

**Fig. 1:**
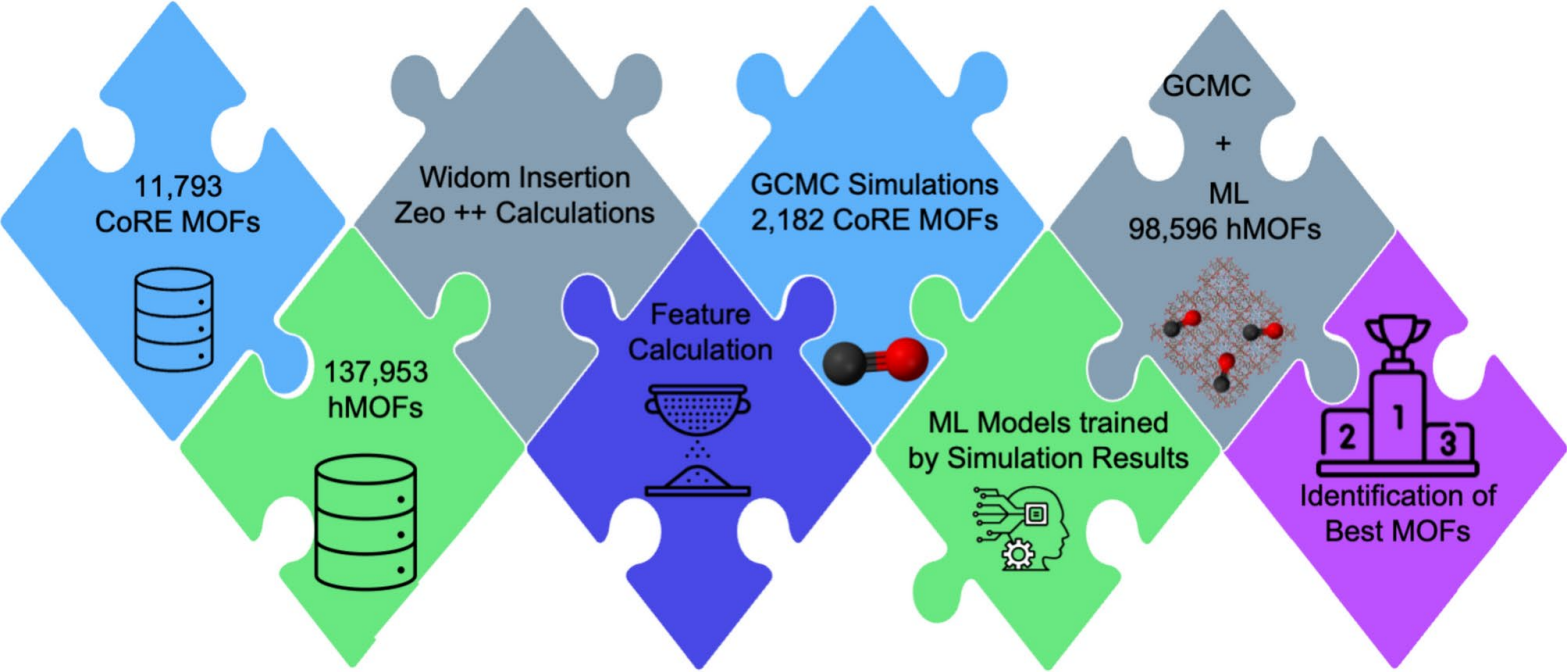
Summary of our computational approach: The MOF library was assembled by considering CoRE MOF and hMOF databases. Various structural, chemical, energy-based descriptors were generated for these MOFs and structural filtering resulted in 2,182 CoRE MOFs and 98,596 hMOFs. GCMC simulations were conducted to compute CO uptakes for 2,182 CoRE MOFs. Using this simulation data along with structural, chemical and energetic features of 2,182 CoRE MOFs, ML models were developed. These ML models predicted the CO uptakes of 98,596 hMOFs, followed by GCMC simulations for 2,884 hMOFs identified as potentially high-performing materials achieving CO uptakes larger than 0.8 mol/kg. The top-performing 20 CoRE MOFs and hMOFs were further analyzed for their linker and metal types.

The computational methodology that we proposed to study MOFs for CO adsorption is illustrated in Fig. 1. We focused on 11,793 non-disordered MOF structures from the CoRE MOF 2019 Database^27,28^ for which all solvents were removed. Zeo ++ software (version 0.3)^38^ was used for calculating the structural properties of CoRE MOFs such as the accessible surface area (S_acc_), porosity (ϕ), the largest cavity diameter (LCD), pore limiting diameter (PLD) and number of open metal sites (OMSs). A probe diameter of 3.72 Å representing the N_2_ molecule was used to compute the surface area, whereas a zero probe was used for calculating the geometric pore volume. To avoid inconsistencies in describing the interaction of gases with unsaturated metal centers using a classical force field^39–41^, CoRE MOFs with OMSs were eliminated. Remaining 4,493 structures were filtered with respect to the kinetic diameter of CO molecule resulting in 2,586 CoRE MOFs having PLD > 3.76 Å^41^. For these structures, partial atomic charges were assigned by using the charge equilibration (Qeq) method^42,43^ as implemented in the RASPA simulation software^44^ and MOFs having partial atomic charges larger than 3 e^-^ and smaller than -3 e^-^ were excluded from the final set following the literature^45^. CoRE MOFs having structural disorders and missing hydrogen atoms were also eliminated from our final material set and we ended up with 2,182 diverse MOF structures.

GCMC simulations were performed for these 2,182 CoRE MOFs using RASPA^44^ simulation package to compute the adsorption of CO gas. 5×10^4^ initialization and 5×10^4^ production cycles were used during the simulations to minimize uncertainties in our calculations. We computed the Henry’s constant (K_H_) and heat of adsorption (Q^0^_st_) for CO at infinite dilution using the Widom insertion technique^46^. The Lennard–Jones (LJ) 12–6 potentials and Coulomb interactions were defined for CO molecules. CO was modeled as rigid with three sites, including one site at the center of the molecule and one site on each side of the molecule^11^. The corresponding parameters for CO model were presented in Table S1. Force field parameters were taken from Dreiding^47^ for the MOF atoms, and for the metal atoms, which are not available in Dreiding, Universal Force Field (UFF)^48^ parameters were used. We note that generic force fields like UFF and DREIDING may fail to accurately assess gas adsorption in MOFs having OMSs because they lack the parameters needed to capture the strong interactions between the OMSs and gases such as CO, H_2_O, CO_2_, and CH_4_^13,36^. To overcome this problem, new or modified force fields were suggested in the literature. For example, Jorge and coworkers^13^ developed a new model for CO to mimic experimental CO adsorption data of HKUST-1, and Wang and coworkers^49^ used the force field optimization approach for modeling CH_4_ adsorption in MOFs with OMSs by using a genetic algorithm. Several high-throughput computational screening studies utilized generic force fields since they are transferable to many different types of MOFs and preferred removing MOFs having OMSs from their large databases before studying gas adsorption^50,51^. Following this literature, we chose to exclude MOFs with OMSs in this study to ensure the reliability and consistency of our computational approach. We note that one limitation of excluding MOFs with OMSs is the potential elimination of promising MOFs offering strong CO adsorption properties. To obtain the cross-interaction parameters, Lorentz-Berthelot mixing rules were used. Ewald summation^52^ was performed to calculate the Coulombic interactions. A cut-off distance of 12 Å was used for the LJ interactions. During simulations, all MOFs were modeled as rigid, to save computational time following the literature^53^. The Peng–Robinson equation of state was used to convert the fugacity to pressure. In GCMC simulations, four different types of moves were considered for CO molecules including translation, reinsertion, rotation and swap. All the details related to move probabilities were given within the input files, accessible from https://github.com/gercakir22/MOFs_for_COstorage. To validate the accuracy of our molecular simulations, we compared our simulated CO uptakes with the previously reported experimental and simulated CO uptake data of various MOFs^11,13,15,20,21^ and showed the good agreement between our simulation results and literature data in Fig. S1.

Relying solely on molecular simulations to determine CO adsorption data across a broad spectrum of MOF materials is computationally demanding. To overcome this challenge, we merged the molecular simulations with ML for accurately delivering CO adsorption data for this extensive range of MOFs. To train the most accurate ML models, we used GCMC simulation data of 2,182 CoRE MOFs along with their 4 structural descriptors (PLD, LCD, S_acc_, and ϕ), 11 chemical descriptors (percentages of carbon, hydrogen, nitrogen, oxygen, halogens, ametals, metals and metalloids in CoRE MOFs, total degree of unsaturation (TDU), the ratio of oxygen atoms to metal atoms (O/M), and the ratio of electronegative atoms to all atoms (E/T)), and an energy-based (Henry’s constant of CO (K_H,CO_)) descriptor. We then analyzed the relationships among these features and their correlation with the simulated CO uptakes calculated at conditions of 0.1, 1, 10 bar, as shown in Fig. S2. We found that Henry’s constant of CO predominantly influences CO uptakes in CoRE MOFs at lower pressures (0.1 and 1 bar). On the other hand, structural descriptors such as pore size, surface area, and porosity play a crucial role in determining CO uptakes at 10 bar, as we will discuss in more detail.

Three different ML models were trained using these descriptors as input data, with the simulated CO uptakes at 0.1, 1, 10 bar as the respective target data. To identify the most suitable algorithms and fine-tune their hyperparameters, we utilized the tree-based pipeline optimization tool (TPOT)^54^ within the auto ML framework. For the selection of the model, the regression algorithms from the scikit-learn^55^ library was used. We implemented the stratified sampling approach to maintain a consistent feature distribution across the test and training data sets, with a denotation of 80% of the data for training and 20% for testing. To avoid overfitting, we implemented fivefold cross-validation. Several statistical accuracies metrics, the coefficient of determination (R^2^), mean absolute error (MAE), root-mean-square error (RMSE), and Spearman’s ranking correlation coefficient (SRCC), as reported in Table S2, were used for evaluating accuracy of the ML models. Given these metrics, TPOT algorithm identified various regression models, specifically GradientBoost^56^ and XGBoost^57^, based on their accuracy to predict CO adsorption data of CoRE MOFs.

In the concluding stage of our study, we evaluated the transferability of our ML models by using them to estimate the CO adsorption data for the whole hMOF dataset at 0.1, 1, and 10 bar. We conducted GCMC simulations for 2,884 hMOFs that exceeded the CO adsorption capacity benchmark. This benchmark was defined by subtracting the largest discrepancy observed between the simulated and ML-predicted CO uptakes of CoRE MOFs included in the test set from the lowest simulated CO uptake among the top 20 CoRE MOFs at 1 bar. We compared ML-predicted and simulated CO uptakes of hMOFs to further affirm the models’ accuracies. Finally, we performed a molecular-level investigation for the top 20 materials from both CoRE MOF and hMOF sets based on their CO uptakes at 1 bar by using MOFSeek^58^ software to reveal the chemical features, such as metal nodes and linker subunit types.

Slow diffusivity of molecules may hinder mass transfer, thereby decreasing the adsorption efficiency of the MOFs^59^. To evaluate the molecular diffusivity in the top CoRE MOFs and hMOFs that we identified, Molecular Dynamics (MD) simulations were performed using the RASPA package^44^ and the self-diffusivity of CO molecules (D_self,CO_) was calculated in the top 20 CoRE MOFs and hMOFs. For MD simulations, CO uptakes obtained from the GCMC simulations at 1 bar, 298 K were used as the initial loadings. For each MD simulation, 4×10^6^ cycles were used with 10^4^ cycles for the initialization and 10^4^ cycles for the equilibration. NVT ensemble was used for simulations using a step size of 1 fs up to a total of 4 ns at 298 K using the Nose–Hoover thermostat^60^. The mean-square displacement of CO molecules was obtained using the modified order-N algorithm implemented in the RASPA, which employs blocking averages to minimize statistical errors. Self-diffusivities were then calculated using Einstein’s relation^61^. Two different trajectories were collected from these simulations to calculate the self-diffusivities of CO molecules.

## Results and discussion

### HTCS and ML results for CoRE MOFs

**Fig. 2:**
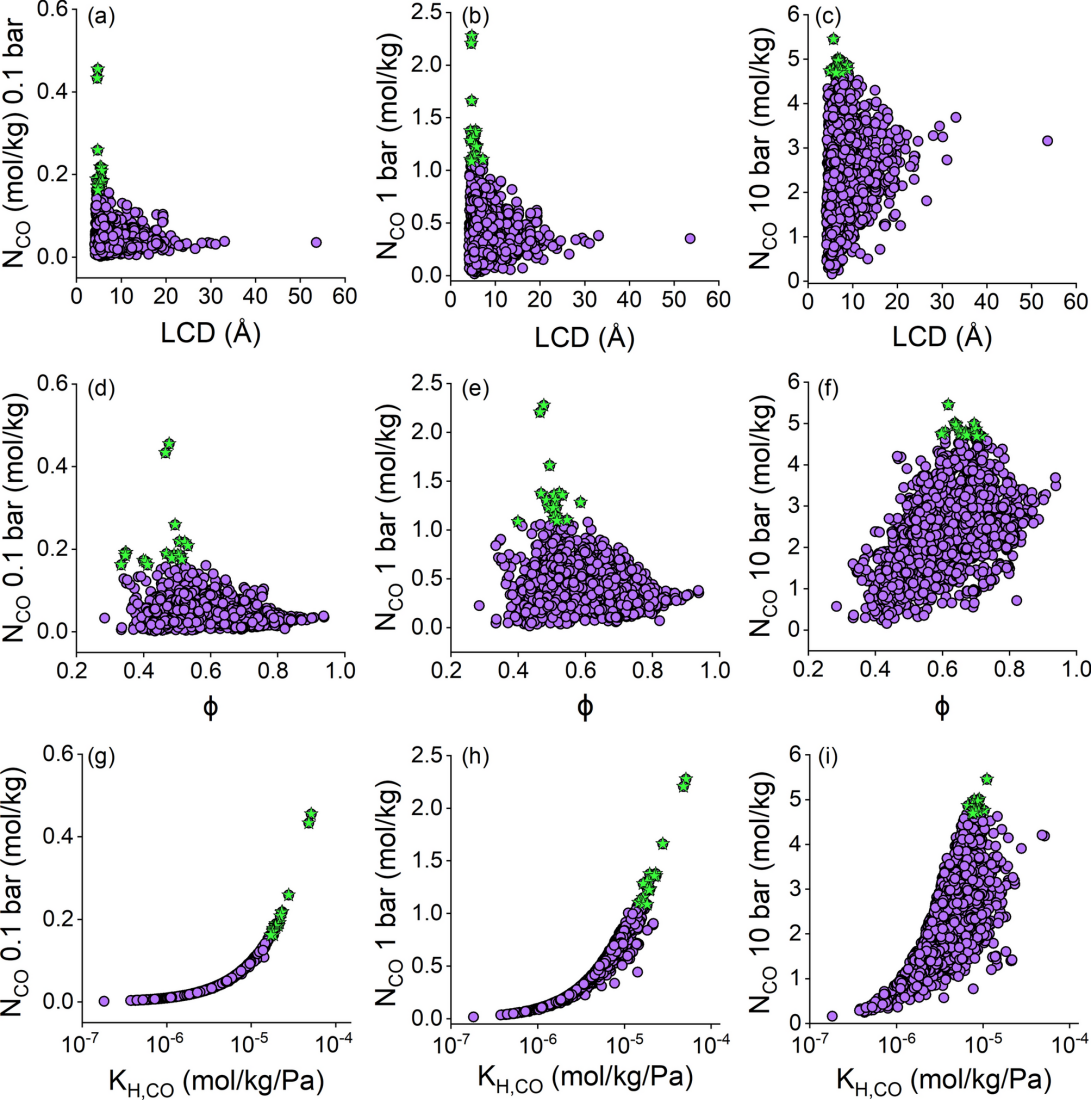
Simulated CO uptakes (N_CO_) of 2,182 CoRE MOFs at 0.1, 1, 10 bar as a function of their (**a**–**c**) largest cavity diameters (LCD), (**d**–**f**) porosities (ϕ), and (**g**–**i**) Henry’s constants of CO (K_H,CO_), respectively. Stars represent the top 20 MOFs having the highest CO uptakes.

Figure. 2 shows the simulated CO uptakes of 2,182 CoRE MOFs computed at 0.1, 1, and 10 bar as a function of their structural (LCD and ϕ) and energy-based (K_H,CO_) descriptors. In Fig. 2a–c, we observed a distinct correlation between pore sizes of materials and their CO uptakes. At lower pressures, we computed CO uptakes in between 1.9×10^–3^-0.46 and 0.02–2.28 mol/kg at 0.1 bar and 1 bar, respectively. At these conditions, CoRE MOFs with LCD < 10 Å can achieve the highest CO uptakes, suggesting that strong confinement of CO molecules in narrow pores can enhance CO adsorption. As pressure increases to 10 bar, we observed a significant widening in the range of CO uptakes (0.16–5.45 mol/kg). Increasing pressure substantially improves CO adsorption, especially for large-pored CoRE MOFs (LCD > 20 Å), leading to high CO uptakes (> 2.5 mol/kg) at high pressures. Figure. 2d and e show that CoRE MOFs with porosities in between 0.4 and 0.7 can achieve CO uptakes larger than 0.2 mol/kg at 0.1 bar, and 1 mol/kg at 1 bar.

In Fig. 2f, we observed that higher porosities contribute to increased CO uptakes larger than 2.5 mol/kg in CoRE MOFs at 10 bar condition. Figure. 2g–i show that there is a strong correlation between K_H,CO_ and CO uptakes at all conditions. CoRE MOFs with high K_H,CO_ consistently have high CO adsorption at all pressures, underlining the importance of MOF-CO interactions. In summary, Fig. 2 indicates that with increasing pressure, structural features (pore size and porosity) play a major role in determining CO uptakes, while at low pressure, adsorbent-gas interactions are more pronounced.

We highlighted the top 20 CoRE MOFs offering the highest CO adsorption at each condition as stars in Fig. 2. These top CoRE MOFs have adsorption capacities of 0.17–0.45, 1.09–2.28, and 4.69–5.45 mol/kg at pressures of 0.1, 1, and 10 bar, respectively, as detailed in Tables S3–S5. These results show that our top MOF candidates outperform several MOFs that we discussed in the literature review section including HKUST-1 (1.16 mol/kg)^18^, MOF-505 (0.97 mol/kg)^18^, CuTDPAT (1.23 mol/kg)^18^, Fe-MIL-100 (0.48 mol/kg)^18^, Cr-MIL-101 (1.09 mol/kg)^18^, MIL-100(Fe) (0.26 mol/kg)^17^, and zeolites 5A (~ 1.2 mol/kg)^15^, 13X (~ 0.5 mol/kg)^15^, and Cu-zeolite Y (1.70 mol/kg)^18^ at 1 bar, 298 K. The top 20 MOFs that we identified at 0.1 and 1 bar generally have narrow pores (4.43–7.21 Å) and low porosities (0.35–0.59), and 14 of them were identified as common top materials at both pressures. At 10 bar, MOFs having larger pores (4.90–8.84 Å) and more porous structures (0.60–0.72) emerge as the top performers. These structures offer more space to accommodate higher numbers of CO molecules.

Our objective was to construct ML models to precisely predict CO adsorption in MOFs, serving as a highly efficient alternative to molecular simulations of thousands of structures. To achieve this, we employed structural and chemical features of 2,182 CoRE MOFs as input data and their simulated CO adsorption results at different pressures as the target data for training ML models. We developed three distinct ML models to predict CO adsorption at three different pressures, as shown in Table S6.

**Fig. 3:**
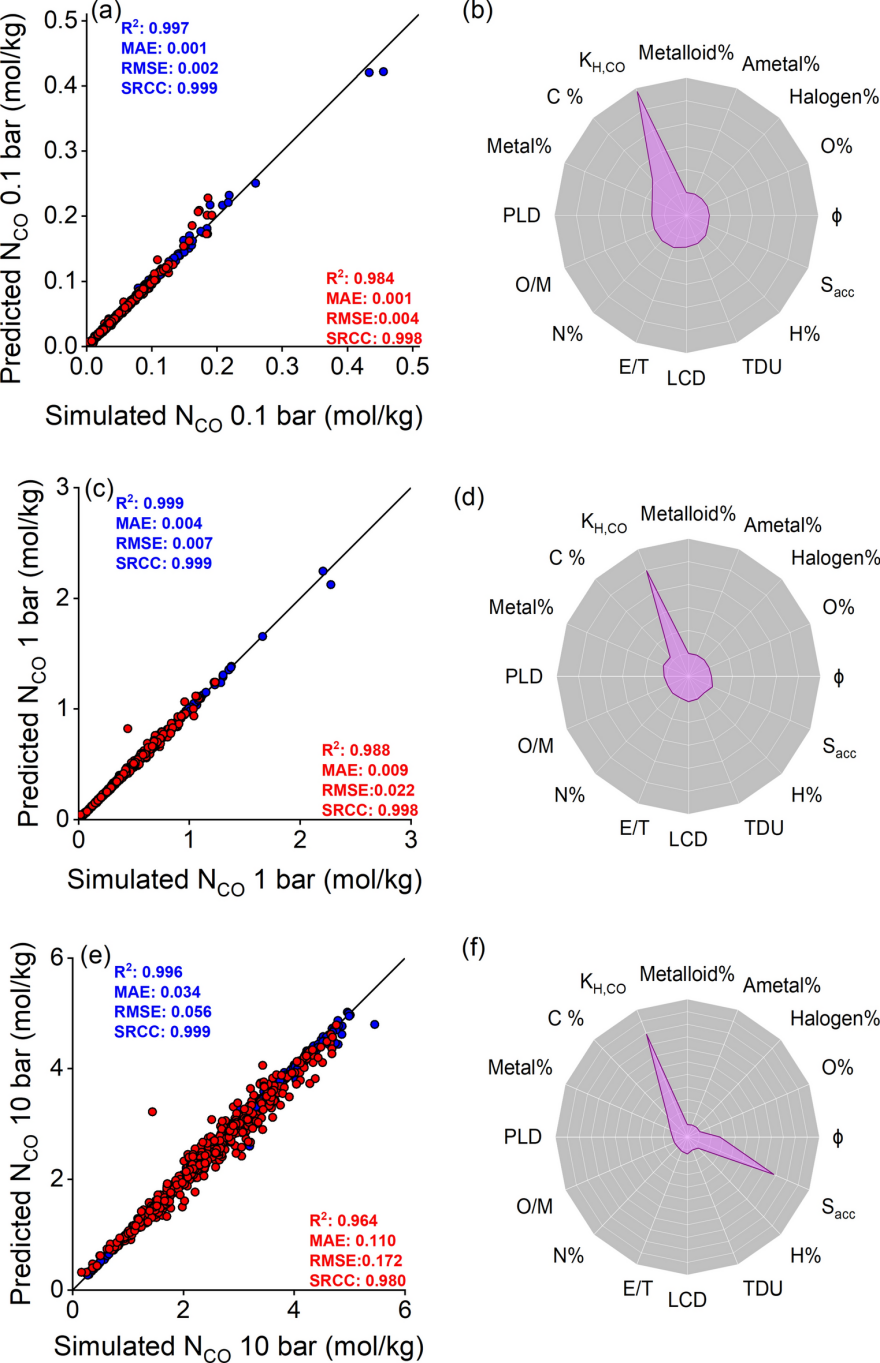
Comparison of the ML-predicted (red and blue represent test and train sets, respectively) and simulated CO adsorption data of 2,182 CoRE MOFs and distributions of the feature importance for our constructed ML models at (**a**,**b**) 0.1 bar, (**c**,**d**) 1 bar, and (**e**,**f**) 10 bar conditions, respectively.

Figure. 3a,c,e show a comparison between ML-predicted CO uptakes with the simulated ones for 2,182 CoRE MOFs. The R^2^ values for the test sets were calculated as 0.984, 0.988, and 0.964 at 0.1, 1, and 10 bar, respectively, demonstrating the strong predictive power of our models. We also observed that our ML models can identify 19, 18, and 15 of the top 20 CoRE MOFs at 0.1, 1, and 10 bar, respectively. Thus, these ML models can be used to accurately identify the most promising CoRE MOFs for CO adsorption. Figure. 3b,d,f represent the distribution of feature importances of our ML models. The Henry’s constant of CO is the most important feature in all our models. Figure. S3 shows that at low pressure (1 bar), its importance value is 0.88. On the other hand, structural features such as surface area (0.34) and porosity (0.07), have an impact on our models at high pressure (10 bar), where importance of Henry’s constant of CO decreases to 0.39.

### ML results for hMOFs

**Fig. 4:**
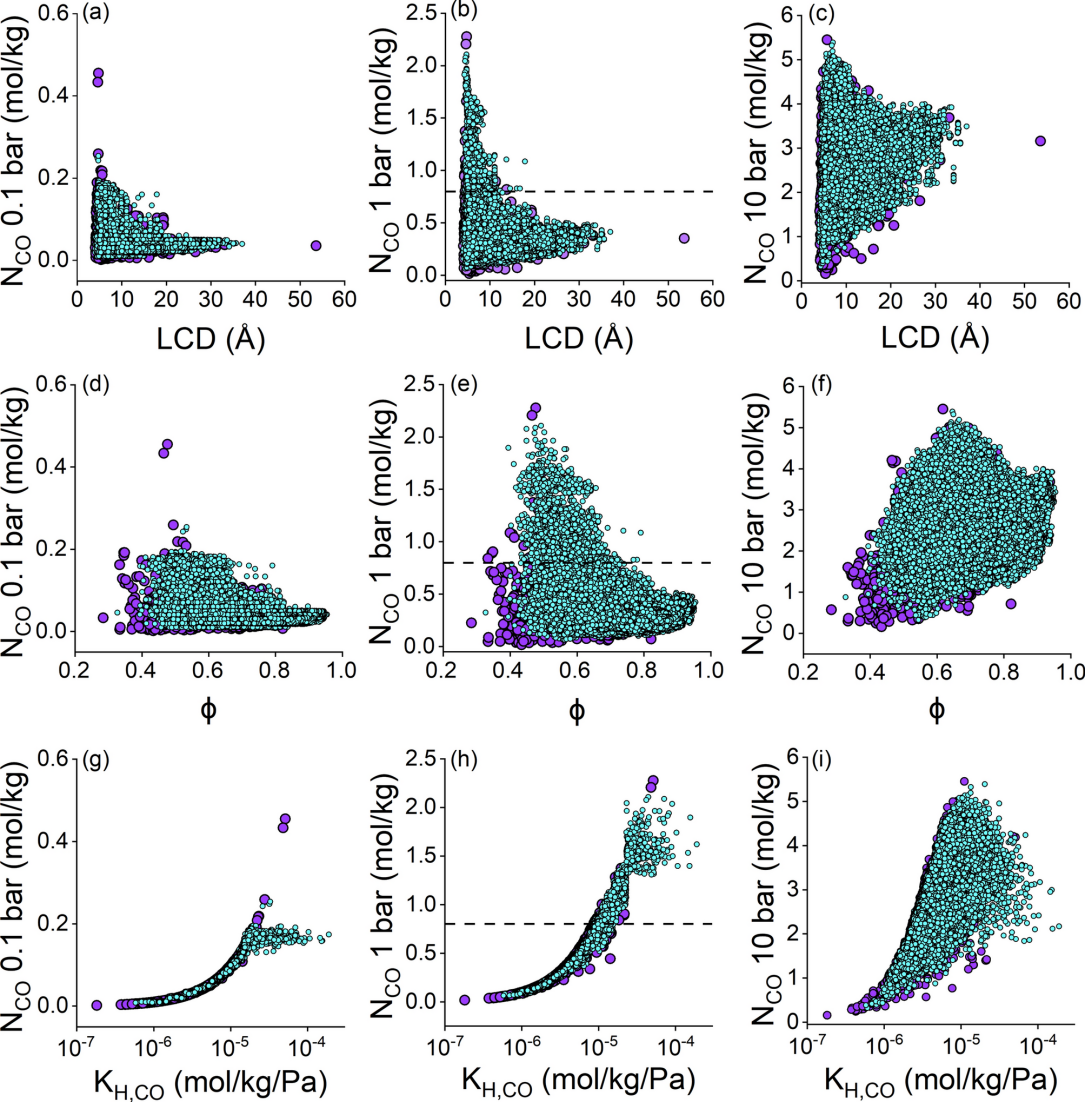
ML-predicted CO uptakes of 98,596 hMOFs (cyan) at 0.1, 1, and 10 bar as a function of their (**a**–**c**) largest cavity diameters (LCD), (**d**–**f**) porosities (ϕ), and (**g**–**i**) Henry’s constants of CO (K_H,CO_). Simulated CO uptakes of 2182 CoRE MOFs (purple) were provided for comparison. The dashed lines in (**b**,**e**,**h**) indicate the minimum threshold (0.8 mol/kg) for high-performing MOFs.

We proceeded to assess the CO adsorption properties of hMOFs by using our ML models. Figure. 4 shows both ML-predicted CO adsorption data of 98,596 different types of hMOFs as a function of materials’ structural and energy-based properties. For comparison, we included the simulated uptakes of CoRE MOFs in the background. The trends observed between ML-predicted CO uptakes and structural properties of hMOFs closely match those observed for the CoRE MOFs, indicating that ML models reliably applied learned knowledge to unseen datasets.

The CO uptakes of hMOFs were predicted in between 0.01–0.25, 0.04–2.20, and 0.41–4.96 mol/kg at 0.1, 1, and 10 bar, respectively. In Fig. 4a–c, we observed that narrow-pored hMOFs (LCD < 10 Å) tend to achieve CO uptakes larger than 0.2 mol/kg and 1 mol/kg at 0.1 and 1 bar, respectively, while MOFs with large pores (LCD > 20 Å) can achieve CO uptakes larger than 3 mol/kg at 10 bar. Figure. 4d–f show that high porosities result in high CO uptakes in hMOFs as adsorption pressure increases, similar to CoRE MOFs. In Fig. 4g–i, the strength of molecular interactions between CO molecules and MOFs, defined by the Henry’s coefficient of CO, is the main factor in determining CO uptakes of hMOFs, especially at 0.1 and 1 bar.

**Fig. 5:**
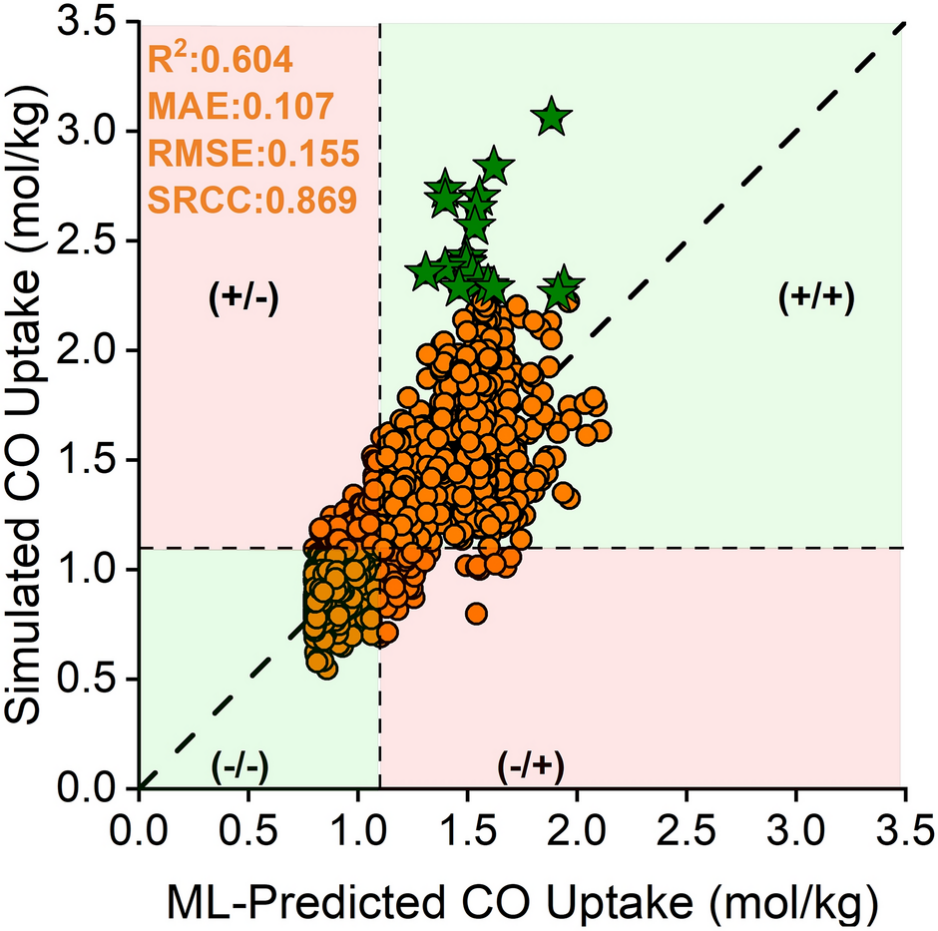
Comparison of the ML-predicted and simulated CO uptakes of 2,884 hMOFs at 1 bar. The green regions represent the true-positive (+ / +) and false negative (-/-); the red regions represent the true-negative (+ /-) and false-positive (-/ +) results. Stars represent the top 20 hMOFs.

Our goal was to identify the top-performing materials among hMOFs for CO adsorption. To achieve this, we set a threshold for the CO uptakes as 0.8 mol/kg based on the CoRE MOF exhibiting the lowest simulated CO uptake (1.09 mol/kg) among the top performers identified at 1 bar and the fluctuations between ML-predicted and simulated CO uptakes in test set of CoRE MOFs (0.3 mol/kg). There are 2,884 hMOFs demonstrating higher CO uptakes than this threshold at 1 bar. It is important to ensure that these high-performing hMOFs can be accurately identified by our ML models amidst potential under/overpredictions. Thus, we computed CO uptakes of these 2,884 hMOFs by performing GCMC simulations at 1 bar and compared the ML-predicted and simulated CO uptakes in Fig. 5.

To evaluate the qualitative predictions of our model, we categorized the results into four: true-positive (+ / +), false-negative (-/-), true-negative (+ /-), and false-positive (-/ +). In this context, ‘true’ indicates a MOF with CO uptake greater than 1.1 mol/kg, while ‘false’ refers to a MOF with CO uptake below this threshold, based on both simulated and ML-predicted values. For instance, if a MOF’s ML-predicted CO uptake exceeds 1.1 mol/kg and molecular simulations confirm this, it is a "true-positive" case. On the other hand, if the ML-predicted CO uptake is below 1.1 mol/kg and the result of molecular simulations agrees, it is a "false-negative" case. In summary, true-positive and false-negative results were considered successful since both the simulation and ML predictions provided the same suggestion. Conversely, true-negative and false-positive results indicated discrepancies between simulation results and ML predictions.

For quantitative analysis, we observed a mediocre R^2^ (0.60), a good ranking score (0.87), and mediocre error values (MAE: 0.11, and RMSE: 0.16) between ML-predicted and simulated CO uptakes for an unseen set of 2,864 hMOFs. During calculation of these metrics, we did not consider top 20 hMOFs identified by molecular simulations. The reason is that we noticed that there is a saturation level in ML-predicted values of the top 20 hMOFs, which is due to the uptake ranges of CoRE MOFs (0.04–2.28 mol/kg) used for model training, and the nature of the regression models which are not able to extrapolate beyond this training data^62^. These top 20 hMOFs exhibit CO uptakes in between 2.26–3.06 mol/kg according to the molecular simulation results, but their uptakes were underpredicted by our ML model, 1.40–1.94 mol/kg.

Our model identified 991 high-performing hMOFs with CO uptakes exceeding 1.1 mol/kg, and 860 of these were validated through molecular simulation results, yielding a success rate of 86.8%. Additionally, our model identified 1,893 hMOFs with moderate performance (0.8–1.1 mol/kg), and 1,660 of these were confirmed by molecular simulations, corresponding to an 87.7% success rate. Notably, all top 20 hMOFs were consistently identified by both our ML model and molecular simulations, highlighting the robustness of our approach in pinpointing the best materials. Although quantitative accuracy of our ML model lays in a mediocre region, it works accurately in a qualitative fashion. Thus, instead of employing a brute-force computational screening approach for thousands of materials via GCMC simulations, our ML-targeted approach provides a meaningful subset of ~ 2% of the whole hMOF material space (2,884 out of 137,953 hMOFs) for discovering the novel hypothetical MOFs outperforming synthesized ones for CO adsorption.

### Analysis of the top materials

**Fig. 6:**
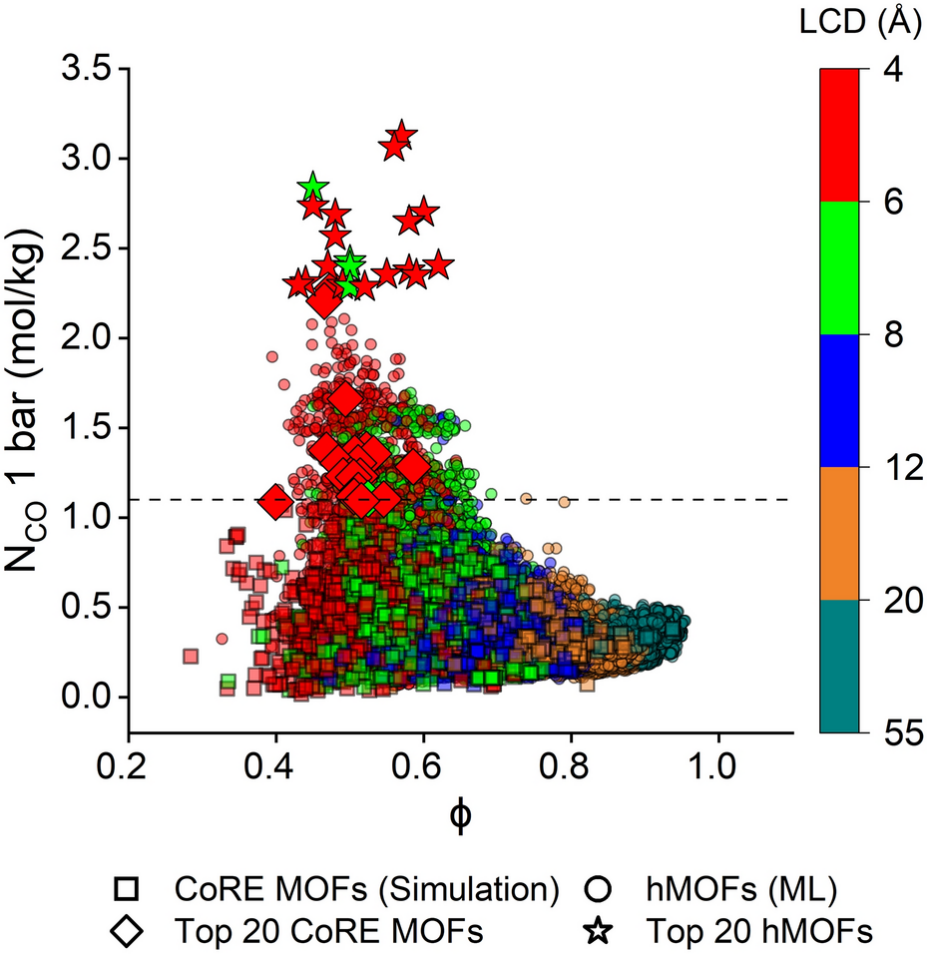
Simulated CO uptakes of 2,182 CoRE MOFs and ML-predicted CO uptakes of 98,596 hMOFs at 1 bar with respect to their porosities and LCDs. Top 20 CoRE MOFs (green stars) and hMOFs (blue stars), identified from the simulation results were also given for comparison and resized to improve eye-tracking when comparing the top-performing CoRE MOFs and hMOFs with the entire MOF material space.

We finally provided a comprehensive overview of our simulation and ML results for both CoRE MOFs and hMOFs, as depicted in Fig. 6. CoRE MOFs characterized by narrow pores and low porosities often demonstrate high CO uptakes. Similarly, ML-predicted CO uptakes for hMOFs follow this trend, indicating that our ML models have effectively discerned the intricate structural-chemical-energetic relationships among the relevant descriptors. As illustrated in Fig. 6, all top-performing CoRE MOFs, represented as diamonds, possess narrow pores (< 8 Å) and low porosities (< 0.65). As depicted in Fig. S4a and b), LCD and porosity distribution of the top 20 MOFs fall within the lower quartile of the entire CoRE MOF set. This suggests that narrow-pored and less porous structures tend to achieve high CO adsorption at 1 bar and 298 K. This is attributed to the pronounced effect of strong gas-MOF interactions at low pressures, which allows CO molecules to be confined within these narrow pores. Figure. S4c illustrates that the top 20 MOFs have higher K_H,CO_ values compared to the average of the K_H,CO_ values of the original CoRE MOF set, indicating that the presence of strong gas-MOF interactions leads to high CO adsorption performance. Correspondingly, among the 1,119 hMOFs with ML-predicted CO uptakes exceeding 1.1 mol/kg, 1,067 (95.3%) of them share these structural features with the top-performing CoRE MOFs, represented as green and red circles. As anticipated, the top-performing hMOFs, depicted as stars, are derived from this subset and exhibit CO uptakes ranging from 2.26 to 3.06 mol/kg, surpassing the top-performing CoRE MOFs with CO uptakes between 1.09 and 2.28 mol/kg. Both the top 20 CoRE MOFs and hMOFs display similar structural attributes, such as narrow pores (LCD: 4.5–7.2 Å) and low porosities (0.40–0.62), as detailed in Tables S4 and S7.

Although top hMOFs and CoRE MOFs have similar structural features, we examined the chemical properties, such as metal types and linker subunits, of these materials to understand why hMOFs exhibit higher CO uptakes compared to CoRE MOFs. In Fig. S5a and b, the analysis of metal and linker subunit types of the top 20 CoRE MOFs revealed that there are 7 different types of metals (Co, Ni, Zn, Cu, Cd, Li, and In), and 12 different linker subunit types as listed with their representations in Fig. S5. An interesting result is that while Co is the most common metal type observed for the top 20 CoRE MOFs, Zn is the most common metal observed for all set. Additionally, rare metals like Li can be found in the top CoRE MOFs. Our findings align with the literature, which shows that MOFs containing Li metals achieve enhanced storage of polar molecules^63^.

For the linker types, except for three, all of them contain an N element, and all linker subunit types contain at least one aromatic ring. Additionally, three of them contains pyridine based linker subunits (L-4, L-8, L-9) which was previously reported for CO capture^64^. The importance of the carboxylic acidic sites for adsorption of polar molecules was also reported in the literature^65^. Similarly, our linker subunits contain carboxylic acid groups (L-1, L-5, L-8 and L-10), and as illustrated in Fig. S5b, 9 of the top 20 CoRE MOFs have carboxylic acid groups. For the top 20 hMOFs, we found that Cu and Zn metals are dominant, and five different linker subunits all containing aromatic rings and nitrogen elements were identified, similar to the linkers found in the top CoRE MOFs. Additionally, eight hMOFs include highly electronegative halogen atoms, such as chlorine.

We also calculated the self-diffusivities of CO molecules in the top CoRE MOFs and hMOFs by performing MD simulations at the adsorbed CO capacities obtained at 1 bar. Tables S4 and S7 show that the self-diffusivities of top MOFs range in between 7.5×10^–6^-8.8×10^–4^ cm^2^/s, while those for top hMOFs vary within 2.1×10^–5^-3.8×10^–4^ cm^2^/s, respectively. Compared to the diffusivity data of previously reported materials such as zeolite 5A (3.4×10^–7^ cm^2^/s) and 13X (1.3×10^–7^ cm^2^/s), MOF-5 (1.3×10^–4^ cm^2^/s) and MOF-177 (2.9×10^–4^ cm^2^/s)^15^, our top-performing CoRE MOFs and hMOFs fall within the same diffusivity range, making them promising adsorbent candidates for CO storage.

## Conclusion

In this study, we examined two different MOF databases, one synthesized and one hypothetical MOF set, for CO adsorption at 0.1, 1, and 10 bar at 298 K. We first conducted an initial screening of a diverse subset of 2,182 CoRE MOF materials, and CO uptakes were calculated between 1.9×10^–3^-0.46 mol/kg, 0.02–2.28 mol/kg, and 0.16–5.45 mol/kg at 0.1, 1, and 10 bar conditions, respectively. We subsequently trained an ML algorithm to accurately predict the CO uptakes of 98,596 hMOFs. Through this ML-assisted computational screening, we identified 2,884 hMOFs that surpassed CO uptake threshold of 0.8 mol/kg at 1 bar and 298 K, aimed at isolating the most efficient hMOF adsorbents. For further investigation, we employed GCMC simulations. A comparison between ML-predicted and simulated CO uptakes demonstrated that (i) our ML model reliably predicts CO uptakes of hMOFs and effectively identifies hMOFs with high CO uptakes, and (ii) the top 20 hMOFs significantly outperform the top 20 synthesized MOFs, exhibiting CO uptake capacities that are 1.5–2 times greater (2.26–3.06 mol/kg for hMOFs versus 1.09–2.28 mol/kg for CoRE MOFs) at 1 bar. Our structural analysis of the top 20 CoRE and hMOFs indicated that MOFs with relatively small pore sizes (< 8 Å) and low porosities (0.4–0.65) can achieve high CO uptakes. Chemical analysis of the top-performing MOFs revealed that structures featuring linker subunits with aromatic rings and carboxylic acid groups, combined with metal nodes like Co, Zn, and Ni, can attain high CO uptakes. We also observed that CO self-diffusivities are in the order of 10^–6^-10^–4^ cm^2^/s, which is similar to diffusivities observed in zeolites and MOFs, and optimal to provide enough contact time between MOF adsorbents and CO molecules for their effective adsorption. Our proposed method significantly accelerates the material screening process, using only a fraction of the computational resources required for analyzing the MOF databases to find the high-performing MOFs by performing GCMC simulations. This method revealed patterns within the MOF material space to aid experimental scientists in identifying high-performing materials for CO storage. We anticipate that the atomic-level understanding of MOF adsorption behavior derived from this study will accelerate the adoption of MOFs as CO adsorbents, thereby considerably progressing initiatives to lower CO emissions.

## Supplementary Information

Supplementary Information.

## Data Availability

Supplementary information is attached in the form of a word document with several additional plots and tables. The data and ML models supporting the findings of this study are available at https://github.com/gercakir22/MOFs_for_COstorage.
