# Publisher Correction: Understanding CO adsorption in MOFs combining atomic simulations and machine learning

**DOI:** 10.1038/s41598-024-79664-w

**Published:** 2024-11-18

**Authors:** Goktug Ercakir, Gokhan Onder Aksu, Seda Keskin

**Affiliations:** Department of Chemical and Biological Engineering, Koç University, Rumelifeneri Yolu, Sariyer, 34450 Istanbul, Turkey

Correction to: *Scientific Reports* 10.1038/s41598-024-76491-x, published online 22 October 2024

In the original version of this article, one reference was omitted from the Reference list.

Reference 25

“Naderlou, S., Vahedpour, M., and Franz, D. M. Exploring the Role of Functional Groups in Modulating NO and CO Adsorption and Diffusion in 2D (Zn)MOF-470: A Multiscale Computational Study. *The Journal of Physical Chemistry C* 2023 *127* (38), 19301-19323 DOI: 10.1021/acs.jpcc.3c05371.”

As a result, the Introduction

“Franz and coworkers focused on CO adsorption in Zn-MOF-470 and its functionalized versions using GCMC simulations https://pubs.acs.org/doi/10.1021/acs.jpcc.3c05371. Results revealed that hydroxyl (-OH) functionalized Zn-MOF-470 achieves the highest uptake 116.2 cm^3^/g (~ 5.18 mol/kg) outperforming others at 1 bar, 298 K.”

now reads,

“Franz and coworkers focused on CO adsorption in Zn-MOF-470 and its functionalized versions using GCMC simulations^25^. Results revealed that hydroxyl (-OH) functionalized Zn-MOF-470 achieves the highest uptake at 116.2 cm^3^/g (~ 5.18 mol/kg) outperforming others at 1 bar, 298 K.”

The original Article has been corrected.

