# Supplementary material for: Understanding CO adsorption in MOFs combining atomic simulations and machine learning
Source: Sci Rep. 2024 Oct 22;14:24931. doi: 10.1038/s41598-024-76491-x (PMC11496673; doi:10.1038/s41598-024-76491-x)
Supplement: Supplementary file 1 — Supplementary Information. [file 41598_2024_76491_MOESM1_ESM.docx]

**Supporting Information**

***for***

**Understanding CO Adsorption in MOFs Combining Atomic Simulations and Machine Learning**

Goktug Ercakir, Gokhan Onder Aksu, Seda Keskin^*^

Department of Chemical and Biological Engineering, Koç University, Rumelifeneri Yolu, Sariyer, 34450, Istanbul, Turkey

Submitted to *Scientific Reports*


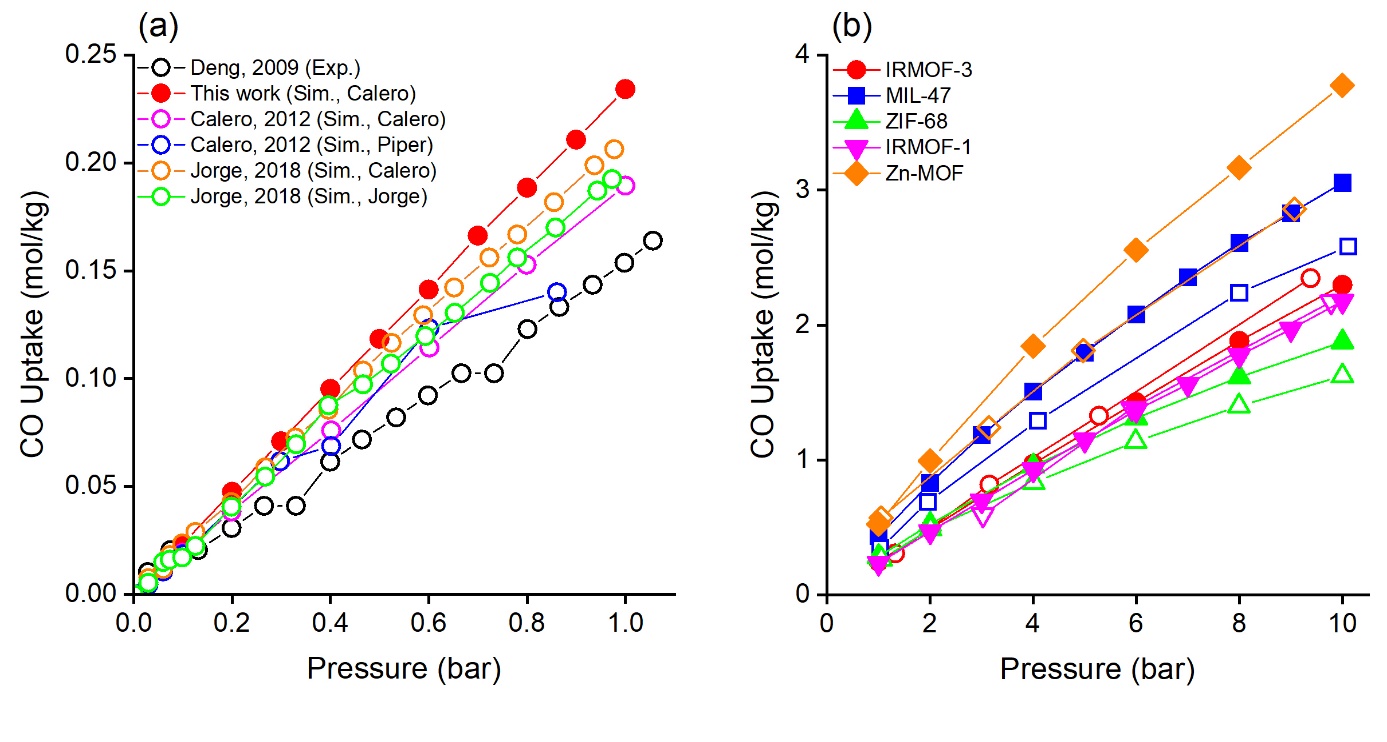


**Figure S1.** (a) Comparison of our simulation results (solid points) with experimentally reported (“Exp.”) CO uptakes and simulated CO uptakes using various CO models (“Sim.”, with the model name) reported in the literature^1-3^ (hollow points) for IRMOF-1 at low pressure (0-1 bar) region. (b) Comparison of our simulation results (solid points) of Zn-MOF^4^ with its experimentally reported CO uptakes, and simulated CO uptakes for IRMOF-3^4^, MIL-47^2^, ZIF-68^5^, IRMOF-1^2^ reported in the literature (hollow points) at high pressure region.

**
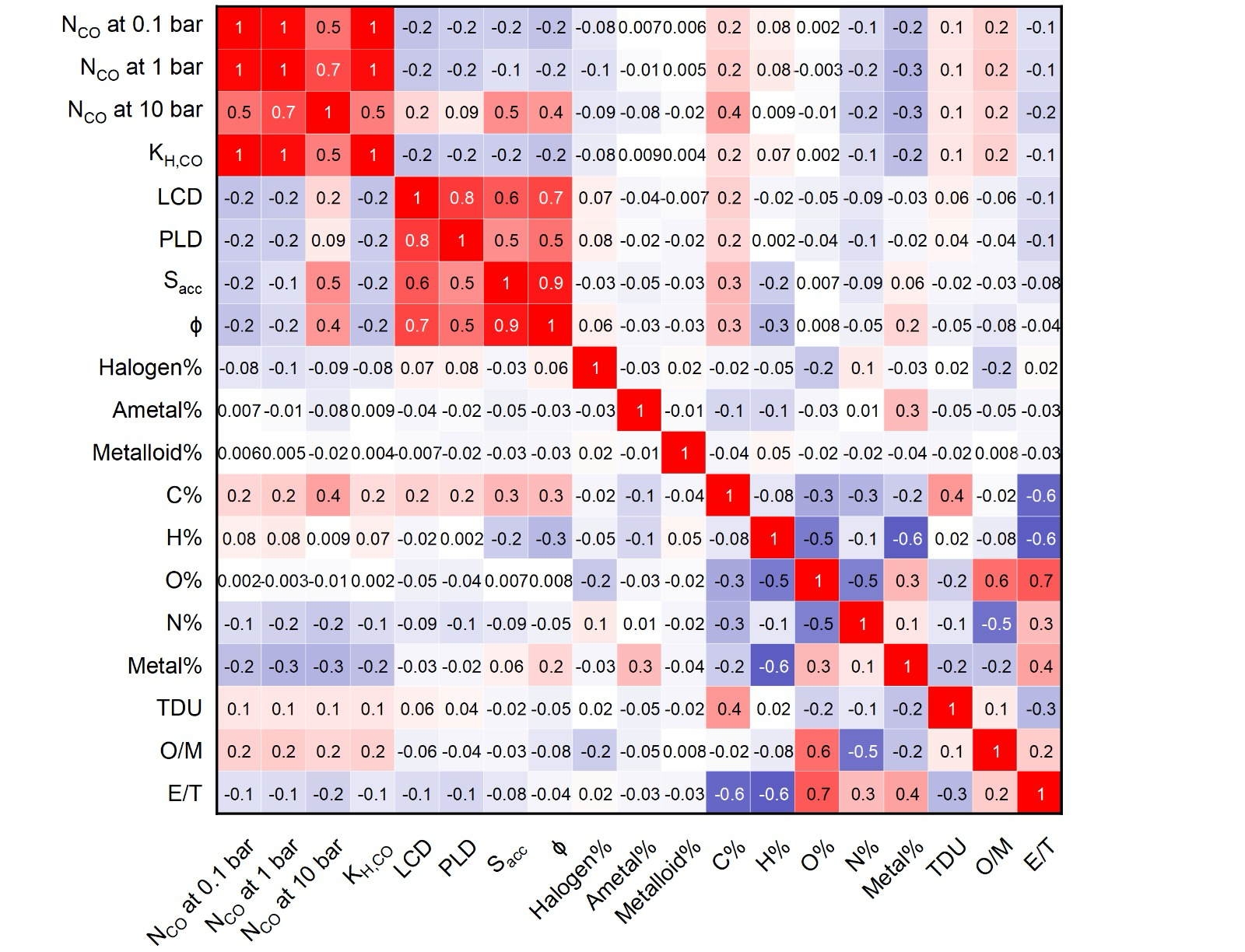
**

**Figure S2.** Pearson correlation plot of all features.

**
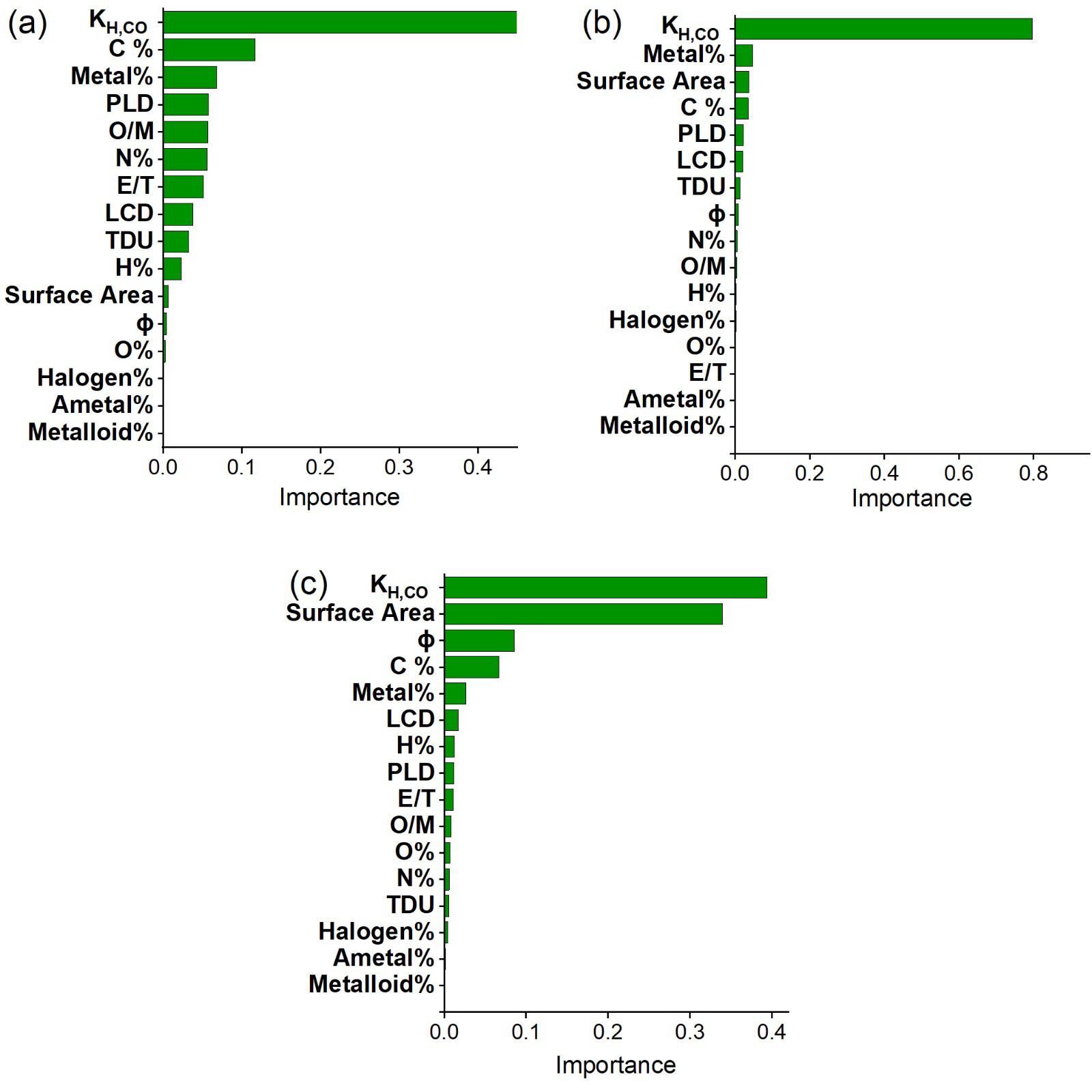
**

**Figure S3.** Distribution of the feature importances in ML models constructed with CoRE MOF simulation data acquired at (a) 0.1, (b) 1, (c) 10 bar, 298 K conditions.


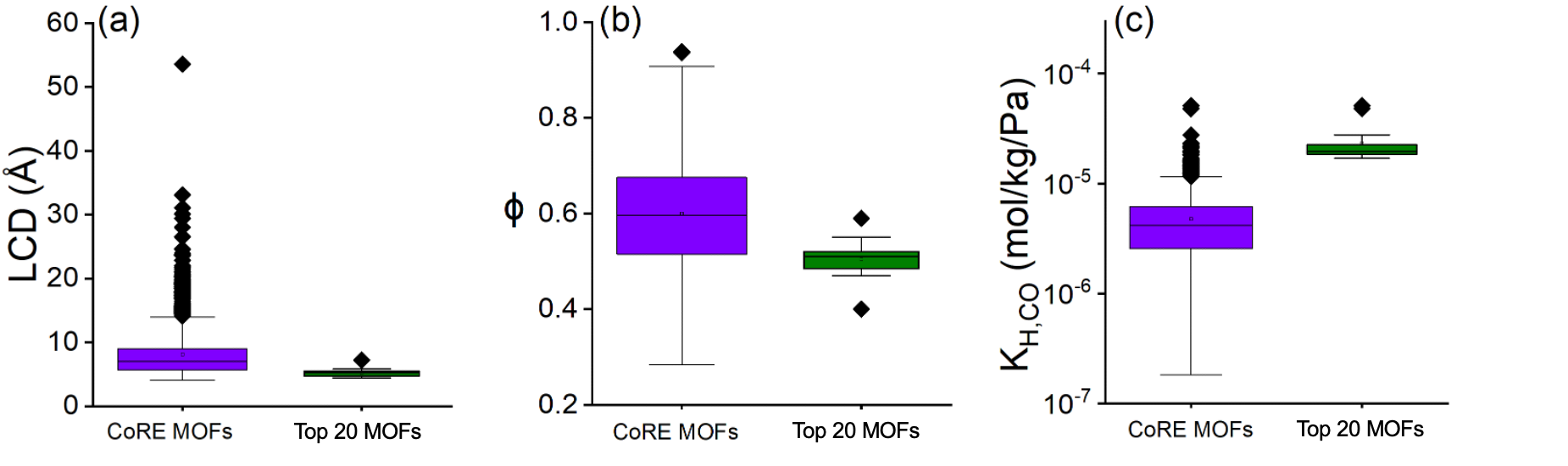


**Figure S4.** Box plots showing the distribution of (a) LCD, (b) ϕ, (c) K_H,CO_ for 2,182 CoRE MOFs and top 20 MOFs at 1 bar, 298 K. Boxes show the quartiles of the dataset, while whiskers extend to show the rest of the distribution, except for the outliers which were defined as values more than 1.5IQR (IQR = interquartile range) from either end of the box.


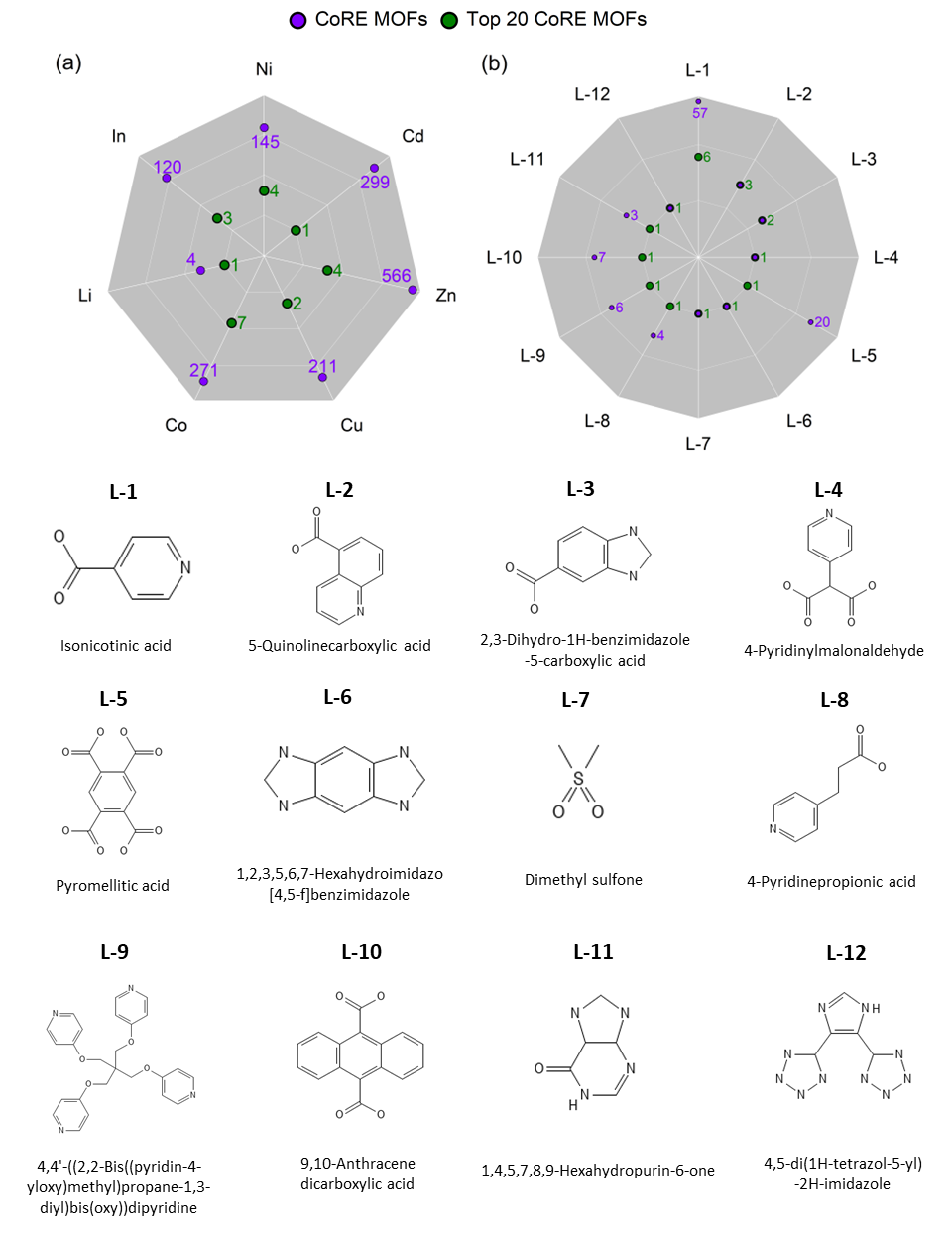


**Figure S5.** The distribution of (a) metal types, and (b) linker subunits among the top 20 CoRE MOFs (green points) and our screened subset consisting of 2,182 CoRE MOFs (purple points). 12 linker subunits identified in the top 20 CoRE MOFs were given with their structures and names.

**Table S1.** Lennard-Jones parameters and partial charges for the model of carbon monoxide considered in this work.

| **Atom type** | **σ (Å)** | **ε/*k*_B_ (K)** | **Charge (*e*)** |
| --- | --- | --- | --- |
| C | 3.636 | 16.141 | –0.2424 |
| O | 2.979 | 98.014 | –0.2744 |
| Dummy | - | - | 0.5168 |

**Table S2**. The statistical accuracy metrics to evaluate the model accuracies.

| Metric | Formula |
| --- | --- |
| Coefficient of Determination (R^2^) | $\text{1-}\frac{\text{1}}{\text{M}}{\sum_{\text{m=1}}^{\text{M}} \left( \bar{\text{y}}\text{-}\hat{\text{y}} \right)}/{\frac{\text{1}}{\text{M}}\sum_{\text{m=1}}^{\text{M}} \left( \text{y-}\hat{\text{y}} \right)^{\text{2}}}$ |
| Mean Absolute Error (MAE) | ${\sum_{\text{m=1}}^{\text{M}} \left\vert\text{y-}\hat{\text{y}} \right\vert}/\text{M}$ |
| Root Mean Square Error (RMSE) | $\sqrt{{\sum_{\text{m=1}}^{\text{M}} \left( \text{y-}\hat{\text{y}} \right)^{\text{2}}}/\text{M}}$ |
| Spearman Ranking Correlation Coefficient (SRCC) | $\text{1-}\frac{\text{6}\sum\text{d}_{\text{i}}^{\text{2}}}{\text{M}\left( \text{M}^{\text{2}}\text{-1} \right)}$ |

M: the number of samples, y: simulated value, $\hat{y}$: predicted value, $\bar{y}$: average of the simulated value, d_i_: the difference between the ranks of corresponding variables.

**Table S3.** CO uptakes, structural and energetic properties of the top 20 CoRE at 0.1 bar.

| MOFs | N_CO_ 0.1 bar (mol/kg) | K_H,CO_ (mol/kg/Pa) | Q^0^_st,CO_  (kJ/mol) | LCD  (Å) | PLD  (Å) | S_acc_ (m^2^/g) | ϕ |
| --- | --- | --- | --- | --- | --- | --- | --- |
| BEDHOJ.P1 | 0.46 | 5.12$\text{×}$10^-5^ | 22.02 | 4.76 | 4.42 | 485.72 | 0.48 |
| PARMIG_clean | 0.43 | 4.80$\text{×}$10^-5^ | 22.48 | 4.62 | 4.28 | 594.26 | 0.47 |
| MIMVEJ_clean | 0.26 | 2.77$\text{×}$10^-5^ | 20.40 | 4.72 | 4.29 | 683.79 | 0.49 |
| GUMDEZ_clean | 0.22 | 2.33$\text{×}$10^-5^ | 18.31 | 5.28 | 4.83 | 865.42 | 0.51 |
| JOXMUG.P1 | 0.22 | 2.29$\text{×}$10^-5^ | 18.25 | 5.59 | 4.51 | 732.46 | 0.52 |
| RITDAB.P1 | 0.21 | 2.21$\text{×}$10^-5^ | 17.98 | 5.59 | 4.57 | 755.36 | 0.53 |
| QUXQUX_clean | 0.19 | 2.16$\text{×}$10^-5^ | 21.59 | 5.17 | 3.83 | 307.03 | 0.35 |
| KAXQIL_clean | 0.19 | 2.14$\text{×}$10^-5^ | 21.85 | 5.12 | 3.77 | 300.86 | 0.35 |
| ADIQEL_clean | 0.19 | 1.96$\text{×}$10^-5^ | 20.42 | 4.43 | 3.91 | 634.72 | 0.47 |
| ARAJOU.P1 | 0.19 | 1.97$\text{×}$10^-5^ | 18.80 | 5.44 | 4.06 | 634.14 | 0.49 |
| ARAJIO.P1 | 0.19 | 1.95$\text{×}$10^-5^ | 18.16 | 5.82 | 4.26 | 729.39 | 0.50 |
| LIDZUV_clean | 0.18 | 2.09$\text{×}$10^-5^ | 21.79 | 5.13 | 3.77 | 294.74 | 0.35 |
| JODGAM.P1 | 0.18 | 1.92$\text{×}$10^-5^ | 17.48 | 5.22 | 4.73 | 808.04 | 0.51 |
| ARAKAH.P1 | 0.18 | 1.94$\text{×}$10^-5^ | 18.23 | 5.79 | 4.26 | 712.15 | 0.51 |
| ic403134c_si_002_clean | 0.18 | 1.83$\text{×}$10^-5^ | 18.80 | 5.08 | 3.78 | 1019.88 | 0.48 |
| UFATEA01.P1 | 0.18 | 1.84$\text{×}$10^-5^ | 18.27 | 5.38 | 4.10 | 645.68 | 0.51 |
| IWELIG01_clean | 0.17 | 1.83$\text{×}$10^-5^ | 21.65 | 4.69 | 4.50 | 273.94 | 0.40 |
| IWELIG_clean | 0.17 | 1.82$\text{×}$10^-5^ | 21.56 | 4.69 | 4.52 | 287.06 | 0.41 |
| QUXRIM_clean | 0.16 | 1.84$\text{×}$10^-5^ | 21.34 | 4.75 | 4.31 | 305.99 | 0.33 |
| IWELOM.P1 | 0.16 | 1.70$\text{×}$10^-5^ | 21.15 | 4.79 | 4.62 | 231.79 | 0.41 |

**Table S4.** CO uptakes, structural and energetic properties of the top 20 CoRE MOFs at 1 bar.

| MOFs | N_CO_ 1 bar (mol/kg) | K_H,CO_ (mol/kg/Pa) | Q^0^_st,CO_  (kJ/mol) | LCD  (Å) | PLD  (Å) | S_acc_ (m^2^/g) | ϕ | D_self,CO_ (cm^2^/s) |
| --- | --- | --- | --- | --- | --- | --- | --- | --- |
| BEDHOJ.P1 | 2.28 | 5.12$\text{×}$10^-5^ | 22.02 | 4.76 | 4.42 | 485.72 | 0.48 | 7.49$\text{×}$10^-4^ |
| PARMIG_clean | 2.21 | 4.80$\text{×}$10^-5^ | 22.48 | 4.62 | 4.28 | 594.26 | 0.47 | 8.79$\text{×}$10^-4^ |
| MIMVEJ_clean | 1.66 | 2.77$\text{×}$10^-5^ | 20.40 | 4.72 | 4.29 | 683.79 | 0.49 | 7.28$\text{×}$10^-4^ |
| JOXMUG.P1 | 1.38 | 2.29$\text{×}$10^-5^ | 18.25 | 5.59 | 4.51 | 732.46 | 0.52 | 1.87$\text{×}$10^-4^ |
| ADIQEL_clean | 1.37 | 1.96$\text{×}$10^-5^ | 20.42 | 4.43 | 3.91 | 634.72 | 0.47 | 6.60$\text{×}$10^-5^ |
| GUMDEZ_clean | 1.36 | 2.33$\text{×}$10^-5^ | 18.31 | 5.28 | 4.83 | 865.42 | 0.51 | 2.85$\text{×}$10^-4^ |
| RITDAB.P1 | 1.36 | 2.21$\text{×}$10^-5^ | 17.98 | 5.59 | 4.57 | 755.36 | 0.53 | 2.46$\text{×}$10^-4^ |
| JODGAM.P1 | 1.30 | 1.92$\text{×}$10^-5^ | 17.48 | 5.22 | 4.73 | 808.04 | 0.51 | 4.70$\text{×}$10^-4^ |
| ic403134c_si_002_clean | 1.30 | 1.83$\text{×}$10^-5^ | 18.80 | 5.08 | 3.78 | 1019.88 | 0.48 | 7.49$\text{×}$10^-6^ |
| EXOFIH_clean | 1.28 | 1.64$\text{×}$10^-5^ | 17.06 | 4.43 | 4.25 | 1225.81 | 0.59 | 2.64$\text{×}$10^-4^ |
| ARAJIO.P1 | 1.23 | 1.95$\text{×}$10^-5^ | 18.16 | 5.82 | 4.26 | 729.39 | 0.50 | 1.49$\text{×}$10^-4^ |
| ARAJOU.P1 | 1.23 | 1.97$\text{×}$10^-5^ | 18.80 | 5.44 | 4.06 | 634.14 | 0.49 | 1.35$\text{×}$10^-4^ |
| UFATEA01.P1 | 1.23 | 1.84$\text{×}$10^-5^ | 18.27 | 5.38 | 4.10 | 645.68 | 0.51 | 7.25$\text{×}$10^-4^ |
| ARAKAH.P1 | 1.22 | 1.94$\text{×}$10^-5^ | 18.23 | 5.79 | 4.26 | 712.15 | 0.51 | 1.36$\text{×}$10^-4^ |
| CEYPUT01_clean | 1.15 | 1.64$\text{×}$10^-5^ | 18.35 | 5.30 | 3.76 | 794.94 | 0.51 | 4.25$\text{×}$10^-5^ |
| CEYPUT_clean | 1.12 | 1.57$\text{×}$10^-5^ | 17.98 | 5.37 | 3.83 | 878.27 | 0.51 | 4.57$\text{×}$10^-5^ |
| UKUCAF_clean | 1.10 | 1.61$\text{×}$10^-5^ | 17.17 | 7.21 | 4.43 | 1243.26 | 0.52 | 5.56$\text{×}$10^-5^ |
| YAQDIF.P1 | 1.10 | 1.46$\text{×}$10^-5^ | 18.26 | 4.45 | 3.82 | 547.74 | 0.55 | 1.48$\text{×}$10^-5^ |
| FUWXOL_SL | 1.09 | 1.65$\text{×}$10^-5^ | 18.07 | 4.77 | 4.43 | 831.54 | 0.52 | 5.80$\text{×}$10^-4^ |
| IWELIG01_clean | 1.09 | 1.83$\text{×}$10^-5^ | 21.65 | 4.69 | 4.50 | 273.94 | 0.40 | 4.56$\text{×}$10^-4^ |

**Table S5.** CO uptakes, structural and energetic properties of the top 20 CoRE MOFs for at 10 bar.

| MOFs | N_CO_ 10 bar (mol/kg) | K_H,CO_ (mol/kg/Pa) | Q^0^_st,CO_  (kJ/mol) | LCD  (Å) | PLD  (Å) | S_acc_ (m^2^/g) | ϕ |
| --- | --- | --- | --- | --- | --- | --- | --- |
| KEVCEV.P1.code | 5.45 | 1.11$\text{×}$10^-5^ | 14.59 | 5.70 | 4.27 | 1984.19 | 0.62 |
| BEPREV01_clean | 5.01 | 9.02$\text{×}$10^-6^ | 12.51 | 6.64 | 5.03 | 3141.59 | 0.64 |
| MURBEI_clean | 4.99 | 7.88$\text{×}$10^-6^ | 11.22 | 7.17 | 5.68 | 2789.93 | 0.69 |
| BEPREV03_clean | 4.98 | 8.73$\text{×}$10^-6^ | 12.25 | 6.75 | 5.17 | 3154.09 | 0.64 |
| BEPREV_clean | 4.96 | 8.69$\text{×}$10^-6^ | 12.25 | 6.75 | 5.16 | 3156.46 | 0.64 |
| HOVQAM_clean | 4.86 | 6.67$\text{×}$10^-6^ | 10.90 | 8.84 | 4.89 | 4918.23 | 0.70 |
| DAFJUS.P1 | 4.86 | 8.03$\text{×}$10^-6^ | 12.33 | 8.75 | 4.58 | 2495.83 | 0.65 |
| KINNAY.P1 | 4.80 | 8.19$\text{×}$10^-6^ | 12.36 | 5.60 | 4.25 | 2745.00 | 0.67 |
| EXEQAA_clean | 4.79 | 9.08$\text{×}$10^-6^ | 13.24 | 7.25 | 7.13 | 1633.14 | 0.61 |
| QUQPOI_SL | 4.79 | 8.08$\text{×}$10^-6^ | 13.41 | 6.68 | 4.17 | 2929.12 | 0.65 |
| CUFDUD_clean | 4.77 | 8.01$\text{×}$10^-6^ | 11.23 | 6.89 | 5.43 | 2720.39 | 0.69 |
| KINNEC.P1 | 4.76 | 8.02$\text{×}$10^-6^ | 12.25 | 5.62 | 4.28 | 2750.87 | 0.67 |
| BEPREV02_clean | 4.76 | 7.79$\text{×}$10^-6^ | 11.50 | 7.19 | 5.45 | 3265.37 | 0.65 |
| DAFJOM_clean | 4.76 | 1.03$\text{×}$10^-6^ | 14.01 | 8.84 | 3.81 | 1744.33 | 0.60 |
| EXEQII_SL | 4.74 | 8.96$\text{×}$10^-6^ | 13.16 | 7.25 | 7.11 | 1642.43 | 0.60 |
| FUYCEJ_clean | 4.73 | 8.94$\text{×}$10^-6^ | 13.30 | 4.90 | 3.78 | 2876.28 | 0.66 |
| RAMPIH.P1.code | 4.71 | 8.24$\text{×}$10^-6^ | 11.90 | 6.18 | 5.49 | 3181.85 | 0.67 |
| ICALOP.P1 | 4.69 | 7.34$\text{×}$10^-6^ | 11.68 | 7.49 | 5.99 | 2915.32 | 0.72 |
| YAMXOC.P1 | 4.69 | 7.61$\text{×}$10^-6^ | 10.84 | 6.24 | 5.54 | 2636.14 | 0.70 |
| CUFDOX.P1 | 4.69 | 7.62$\text{×}$10^-6^ | 10.86 | 6.25 | 5.53 | 2618.01 | 0.70 |

**Table S6.** The ML models constructed by descriptors and their hyperparameters based on the gas adsorption properties of MOFs at 0.1, 1 and 10 bar 298 K.

| **Condition** | **Best Pipeline with Parameters** |
| --- | --- |
| 0.1 bar | XGBRegressor(colsample_bytree=0.8, gamma=0, learning_rate=0.1, max_depth=8, min_child_weight=1, n_estimators=200, nthread=-1, reg_alpha=0.5, reg_lambda=1.0, subsample=0.9, verbosity=0)) |
| 1 bar | GradientBoostingRegressor(alpha=0.99, learning_rate=0.1, loss="huber", max_depth=6, max_features=0.9500000000000001, min_samples_leaf=1, min_samples_split=6, n_estimators=100, subsample=0.45) |
| 10 bar | GradientBoostingRegressor(alpha=0.95, learning_rate=0.1, loss="huber", max_depth=7, max_features=0.9, min_samples_leaf=1, min_samples_split=5, n_estimators=100, subsample=0.55) |

**Table S7.** CO uptakes, structural and energetic properties of the top 20 hMOFs at 1 bar.

| MOFs | N_CO_ 1 bar (mol/kg) | K_H,CO_ (mol/kg/Pa) | LCD  (Å) | PLD  (Å) | S_acc_ (m^2^/g) | ϕ | D_self,CO_ (cm^2^/s) |
| --- | --- | --- | --- | --- | --- | --- | --- |
| hMOF-5081773-(id_139936) | 3.06 | 6.91$\text{×}$10^-5^ | 4.72 | 3.87 | 1764.69 | 0.56 | 3.56$\text{×}$10^-4^ |
| hMOF-35246-(id_50152) | 2.84 | 1.89$\text{×}$10^-4^ | 6.40 | 5.83 | 548.14 | 0.45 | 2.04$\text{×}$10^-5^ |
| hMOF-24784-(id_39807) | 2.73 | 1.52$\text{×}$10^-4^ | 4.88 | 4.48 | 634.15 | 0.45 | 5.79$\text{×}$10^-5^ |
| hMOF-5057125-(id_116154) | 2.70 | 5.87$\text{×}$10^-5^ | 5.14 | 4.40 | 1733.87 | 0.60 | 3.87$\text{×}$10^-4^ |
| hMOF-35234-(id_50142) | 2.69 | 1.07$\text{×}$10^-4^ | 5.05 | 4.38 | 668.36 | 0.48 | 8.59$\text{×}$10^-5^ |
| hMOF-5081749-(id_139913) | 2.65 | 5.56$\text{×}$10^-5^ | 5.08 | 3.88 | 1684.34 | 0.58 | 3.21$\text{×}$10^-4^ |
| hMOF-35243-(id_50153) | 2.57 | 1.45$\text{×}$10^-4^ | 5.01 | 4.47 | 621.61 | 0.48 | 1.25$\text{×}$10^-4^ |
| hMOF-24748-(id_39772) | 2.43 | 6.66$\text{×}$10^-5^ | 7.32 | 6.37 | 674.60 | 0.50 | 7.79$\text{×}$10^-5^ |
| hMOF-5081375-(id_139539) | 2.40 | 4.74$\text{×}$10^-5^ | 5.96 | 4.51 | 2052.72 | 0.62 | 3.99$\text{×}$10^-5^ |
| hMOF-5035421-(id_95001) | 2.40 | 8.28$\text{×}$10^-5^ | 5.46 | 4.49 | 745.64 | 0.47 | 3.69$\text{×}$10^-5^ |
| hMOF-24750-(id_39775) | 2.39 | 7.30$\text{×}$10^-5^ | 6.73 | 6.32 | 663.58 | 0.50 | 7.34$\text{×}$10^-5^ |
| hMOF-5054165-(id_113201) | 2.37 | 4.16$\text{×}$10^-5^ | 5.55 | 4.72 | 1593.59 | 0.58 | 1.15$\text{×}$10^-4^ |
| hMOF-5081391-(id_139556) | 2.35 | 4.87$\text{×}$10^-5^ | 5.20 | 4.21 | 1131.85 | 0.55 | 3.82$\text{×}$10^-5^ |
| hMOF-5057124-(id_116155) | 2.35 | 3.79$\text{×}$10^-5^ | 5.14 | 4.46 | 1698.67 | 0.59 | 4.87$\text{×}$10^-5^ |
| hMOF-5060577-(id_119376) | 2.31 | 7.78$\text{×}$10^-5^ | 5.37 | 4.35 | 627.97 | 0.44 | 7.64$\text{×}$10^-5^ |
| hMOF-5060579-(id_119379) | 2.30 | 8.83$\text{×}$10^-5^ | 5.32 | 3.87 | 553.96 | 0.43 | 2.31$\text{×}$10^-5^ |
| hMOF-35171-(id_50080) | 2.30 | 8.18$\text{×}$10^-5^ | 4.73 | 4.41 | 650.83 | 0.49 | 4.15$\text{×}$10^-5^ |
| hMOF-24757-(id_39779) | 2.29 | 5.23$\text{×}$10^-5^ | 7.01 | 6.24 | 801.14 | 0.50 | 8.05$\text{×}$10^-5^ |
| hMOF-24178-(id_39202) | 2.29 | 5.27$\text{×}$10^-5^ | 4.74 | 4.36 | 871.42 | 0.52 | 1.77$\text{×}$10^-4^ |
| hMOF-35173-(id_50079) | 2.26 | 7.91$\text{×}$10^-5^ | 4.73 | 4.39 | 620.41 | 0.49 | 3.94$\text{×}$10^-4^ |

**References**

1 Saha, D. & Deng, S. Adsorption Equilibria and Kinetics of Carbon Monoxide on Zeolite 5A, 13X, MOF-5, and MOF-177. *J. Chem. Eng. Data* **54**, 2245-2250 (2009).

2 Martín-Calvo, A., Lahoz-Martín, F. D. & Calero, S. Understanding Carbon Monoxide Capture Using Metal–Organic Frameworks. *J. Phys. Chem. C* **116**, 6655-6663 (2012).

3 Campbell, C., Gomes, J. R., Fischer, M. & Jorge, M. New Model for Predicting Adsorption of Polar Molecules in Metal–Organic Frameworks with Unsaturated Metal Sites. *J. Phys. Chem. Lett.* **9**, 3544-3553 (2018).
